# Social and Psychological Readiness to Take Collective Action Against Violence Against Women: A Mixed Methods Study of Informal Settlements in Mumbai, India

**DOI:** 10.1177/1077801220971360

**Published:** 2020-11-23

**Authors:** Lu Gram, Proshant Chakraborty, Nayreen Daruwalla, David Osrin

**Affiliations:** 1 University College London, UK; 2 Society for Nutrition, Education and Health Action, Mumbai, India

**Keywords:** community mobilization, collective action, community readiness, India, domestic violence

## Abstract

Past failures to mobilize communities in collective action against violence against women (VAW) have been ascribed to contextual challenges, but researchers have not systematically mapped community capacity for collective action against VAW. We conducted a mixed methods study in Mumbai, India using quantitative data from a household survey (*n* = 2,642) and qualitative data from 264 community meetings. We found attitudes supporting gender inequality and violence coexisted with significant enthusiasm and support for collective action against VAW. These findings open up avenues for policymakers to treat communities as less vulnerable and more capable of changing situations and problems that affect them.

## Introduction

Violence against women (VAW) is a critical public health and human rights concern with severe human and economic costs. One form of VAW, physical or sexual intimate partner violence, affects 30% of women worldwide (Devries et al., 2013; Garcia-Moreno & Watts, 2011). Intimate partner violence is often sustained by mutually reinforcing drivers of economic vulnerability and patriarchal gender norms (Gibbs et al., 2018), and is an important cause of mental, physical, sexual, and reproductive harm to women (Devries et al., 2013; Dillon et al., 2013; Hill et al., 2016). International declarations—including the Sustainable Development Goals—have committed national governments to eliminating VAW (United Nations [UN], 2017), but investments in prevention and services for survivors of violence remain inadequate (Garcia-Moreno & Watts, 2011).

Interventions that mobilize communities to tackle the social and structural drivers of violence are some of the most effective known to prevent VAW (Bourey et al., 2015). For example, community-based interventions in Uganda and South Africa have successfully reduced rates of violence by training volunteer activists to promote anti-VAW messages, organizing community-wide campaigns and marches, and convening regular meetings of lay women and men to stimulate reflection and action (Abramsky et al., 2014; Pronyk et al., 2006; Wagman et al., 2015). Recent randomized controlled evaluations in Rwanda, Afghanistan, India, and Nepal, however, have failed to show similar effects and this raises questions about transferability between contexts (Chatterji et al., 2020; Clark et al., 2020; Gibbs et al., 2020; Holden et al., 2016; Jejeebhoy & Santhya, 2018).

Community mobilization interventions have generally adopted a phased approach to intervention implementation, consisting of (a) community entry and assessment to establish relationships with stakeholders and map out community resources, (b) awareness-raising activities around gender inequality and VAW as issues for communities to take seriously, (c) capacity-building activities to help communities take action, and (d) consolidation and institutionalization of community-based activities to ensure long-term sustainability (Kyegombe et al., 2014; Michau, 2007; Wagman et al., 2012). These phases correspond approximately with those of the Community Readiness Model, which has long been applied in high-income contexts to match health interventions with communities depending on their baseline level of awareness and engagement with a health issue (Edwards et al., 2000).

When evaluations of community mobilization interventions have failed to show an impact on violence, researchers have often suggested that entrenched gender inequality, chronic poverty, or poor baseline social cohesion made impact infeasible within the allotted timespan (Chatterji et al., 2020; Clark et al., 2020; Hatcher et al., 2020). Under such conditions, implementers may have to spend so much time on awareness-raising that they have insufficient time to implement other forms of action against VAW (Chatterji et al., 2020). Successful interventions have been described as “intentionally generating” social capital to address VAW (Pronyk et al., 2008), which evokes images of communities as blank slates upon which external agents inscribe new social relations and gender attitudes.

Little research has systematically mapped the extent to which residents are actually willing to take action to address VAW in a low- and middle-income context before large-scale public health interventions are introduced. Process evaluations of violence prevention programs have described how communities have responded to interventions, but have not assessed their readiness to take action before the intervention was introduced (Abramsky et al., 2016; Hargreaves et al., 2009; Hatcher et al., 2020). Studies of social capital, social disorganization, and VAW outside the context of intervention evaluations have been mainly carried out in high-income contexts (Beyer et al., 2015; Pinchevsky & Wright, 2012; VanderEnde et al., 2012). Other studies have explored demographic correlates of activism to prevent VAW (Abramsky et al., 2018), and their impact on knowledge, behavior, and relationship quality (Cislaghi et al., 2019; Starmann et al., 2018), but have not measured social or psychological drivers of activism.

To address this evidence gap, we evaluated social and psychological readiness for collective action prior to the introduction of a complex community intervention to prevent VAW in Mumbai, India (Daruwalla, Machchhar, et al., 2019). Drawing on theories of participation in collective action for environmental and political causes (Gram et al., 2019), we developed an analytical framework and validated a scale for measuring the social and psychological drivers of collective action to address VAW (Gram et al., 2020). Our research question was, to what extent are residents of urban informal settlements in our context ready to engage in collective action against VAW?

## Analytical Framework

**Figure 1.**
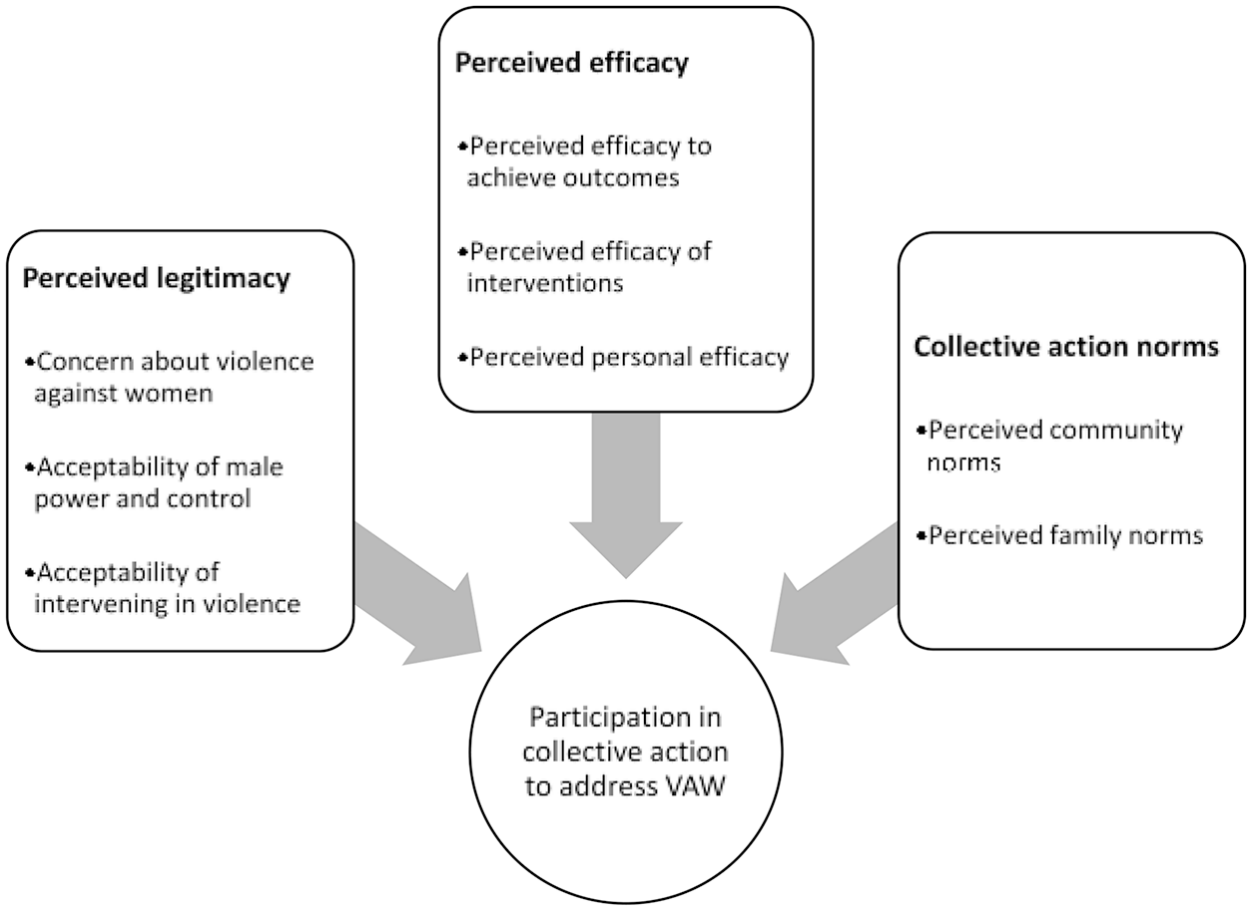
Analytical framework for conceptualizing readiness to take action to address violence against women (VAW) (from Gram et al., 2020).

We define collective action as voluntary joint action by a group of people in pursuit of a shared goal (Meinzen-Dick et al., 2004). It overlaps with, but differs from, the related concept of “bystander intervention” (McMahon & Banyard, 2012) by emphasizing intentional participation in a collective effort rather than spontaneous crisis response by individuals. We operationally define “readiness” to take collective action against VAW as the extent to which enabling drivers of such action are present in a given community. The overall analytical framework is shown in Figure 1 (Gram et al., 2020).

*Perceived legitimacy* refers to the extent to which action against VAW is seen as acceptable. Multiple social science theories have proposed that perceived grievance, injustice, or deprivation can motivate collective action for social change (Van Zomeren et al., 2008). Conversely, activists and political theorists have long held that prevailing societal myths serving to legitimize the powerful may lead individuals to take action contrary to their own collective interests (Freire, 1972; Jost et al., 2004; Radke et al., 2016). With regard to action against VAW, we conceptualized the construct as composed of subconstructs denoting respondent concern about VAW in general, acceptance of male power and control in the household, and beliefs about the acceptability of intervening in actual cases of VAW.

*Perceived efficacy* refers to the extent to which participation in collective action is seen as a successful approach to addressing VAW. This construct aligns with theories positing that individuals need to feel their participation is potentially impactful before they decide to take action (Hornsey et al., 2006; Van Zomeren et al., 2008). It also aligns with ideas that self-efficacy, perceived competence, and self-confidence influence participation in civic affairs (Peterson & Zimmerman, 2004; Zimmerman, 1995; Zimmerman & Rappaport, 1988). We divided the construct into subconstructs: perceived efficacy to achieve specific outcomes (e.g., get the police to take action), perceived efficacy of specific interventions (e.g., group discussions), and perceived contribution of individuals’ own participation.

*Collective action norms* refer to the extent to which community members expect others to approve or disapprove of them taking action to address VAW. This construct aligns with theories that social norms imposing rewards for participation and penalties for nonparticipation are needed to produce collective action (Olson, 1971; Ostrom, 1998). It also aligns with theories that such social norms may actively discourage or stigmatize it (Jordan & Maloney, 2006; Lodhia, 2014; Radke et al., 2016). We divided this construct into two subconstructs concerning respondent perceptions of the reaction of family and community members to their participation in collective action against VAW.

## Method

### Study Context

Our study was embedded in an ongoing cluster-randomized controlled trial evaluating the effects of a complex community intervention to prevent VAW implemented by the non-government organization SNEHA (Society for Nutrition, Education and Health Action) in Mumbai, India (Daruwalla, Machchhar, et al., 2019). We consciously conducted our study while the broader evaluation was ongoing, to avoid post hoc rationalization of trial results. The main beneficiaries of the SNEHA program on prevention of violence against women and children are residents of informal settlements, characterized by overcrowding, insubstantial housing, insufficient water and sanitation, lack of tenure, and hazardous location (UN-Habitat, 2003). Worldwide, one in eight people reside in informal settlements (UN-Habitat, 2016), and they constitute 41% of Mumbai households (Chandramouli, 2011). The intervention engaged community organizers in convening groups of women, men, and adolescents over a 3-year period to address VAW on a platform of existing counseling, therapy, and legal services. The primary outcomes were the prevalence of physical or sexual domestic violence and the prevalence of emotional or economic domestic violence, control, or neglect, both in the preceding 12 months. Secondary outcomes included non-partner sexual violence.

### Methodological Approach

We used a mixed methods approach to add depth to our understanding of collective action that could not be easily obtained through independent quantitative or qualitative studies. We used a convergent design in which quantitative and qualitative data were collected independently, but analyzed together, known as integration at the level of interpretation (Fetters et al., 2013). We triangulated between quantitative and qualitative data to assess the validity of our findings and enhance the richness of their interpretation and explanation (Fetters et al., 2013). Data collection took place from December 2017 to December 2019.

### Quantitative Data Collection

We carried out a baseline survey in 54 trial clusters of around 500 households in four large urban informal settlements. Investigators selected women and men aged 18 to 65 years to interview—one per household—and visited households sequentially. The survey comprised questions on attitudes to gender roles, gender equality, VAW, and bystander intervention, as described in the protocol (Daruwalla, Machchhar, et al., 2019). Questions on action to address VAW were added later, resulting in 92 respondents missing data. After dropping these (3%), the final sample size was 2,642, of whom 1,307 were cis men, 1,331 cis women, and four trans women. Details of data collection and validation of the survey items have been reported elsewhere, and analysis of missing data did not show appreciable bias (Gram et al., 2020).

### Qualitative Data Collection

We collected baseline data from 24 of 27 intervention clusters. The COVID-19 pandemic prevented us from collecting data in the last three clusters. We trained a community team in conducting micro-planning exercises (Daruwalla et al., 2015) to carry out a rapid needs assessment of community concerns regarding issues of VAW. Community team members facilitated 11 sessions per cluster, with each session lasting between 60 and 90 min, resulting in a total of 264 sessions across 24 clusters. Discussions focused on the prevalence and seriousness of VAW, safety, household conflict, women’s mobility, and support services for survivors. Community team members wrote brief descriptions of each session using open-ended templates. Proshant Chakraborty (PC) and qualitative researcher Apoorwa Gupta (AG) conducted participant observation in 120 of 264 sessions and had informal discussions with participants after each session. They recorded the responses in written field notes, which they later transcribed into Microsoft Excel.

### Data Analysis

We first conducted quantitative and qualitative analysis separately. Lu Gram (LG) did a descriptive analysis of quantitative data by inspecting crude percentages and frequencies of each indicator disaggregated by gender. As there were only four transwomen in the sample, we grouped them with cis-gender women. We tested for gender differences using Pearson chi-square tests. PC and AG reviewed their own field notes and reports from the community team. They coded all the qualitative data and developed the codes into themes using thematic analysis (Saldaña, 2015). They discussed these emergent themes throughout with Nayreen Daruwalla (ND) and David Osrin (DO) to reduce the influence of their own position and stimulate reflective analysis.

LG and PC integrated the qualitative and quantitative results using a narrative “weaving approach” (Fetters et al., 2013), in which qualitative and quantitative findings were analyzed together using our analytical framework rather than separately. We looked for points at which both types of data confirmed, expanded upon, or disagreed with one another. However, given the same qualitative theme often related to multiple quantitative indicators in different parts of our analytical framework, we decided to present quantitative and qualitative results in different sections. We presented our results to the SNEHA intervention team to elicit their feedback as a form of member check.

### Ethics

The trial and associated data collection were approved by the Institutional Ethics Committee of Partners for Urban Knowledge, Action, and Research (PUKAR) (25 December 2017), and the University College London Research Ethics Committee (3546/003, 27 September 2017). The TARA trial within which data collection took place is registered with the Controlled Trials Registry of India (CTRI/2018/02/012047, 21 February 2018) and with ISRCTN84502355 (22 February 2018: http://www.isrctn.com/ISRCTN84502355). We followed World Health Organization (WHO) guidelines for research on domestic violence against women (World Health Organization, 2001). Survey interviewers provided participant information sheets to respondents, discussed the nature of the interview, and obtained signed consent.

## Results

### Quantitative Results

#### Participation in collective action

We found collective action to be common (Table 1). Forty-six percent of women and 62% of men had witnessed a march, rally, or protest in the past year and 21% of women and 28% of men had regularly attended meetings of a local community-based group or non-governmental organization in the past year. Thirteen percent of men and 12% of women had attended a group meeting or gathering addressing VAW as an issue. Men were more likely overall than women to participate in local groups or organizations. When women participated, they participated disproportionately in economic self-help groups compared with men, whereas men were much more likely to participate in political, caste, or religious groups.

**Table 1.** Participation in Collective Action to Address Violence Against Women.

Indicator	Women and trans women		Men		χ^2^-test of differences
	Yes % (*n*)	No % (*n*)	Yes % (*n*)	No % (*n*)	*p*
Regularly attended meetings of community-based group in past 12 months	18 (238)	82 (1,097)	25 (324)	75 (983)	<.001
Regularly attended meetings of non-government organization in past 12 months	8 (101)	92 (1,234)	9 (120)	91 (1,187)	.134
Regularly attended meetings of either community-based group or non-government organization in past 12 months					
*(If yes)* Type of CBG/NGO . . .^a^	21 (283)	79 (1,052)	28 (364)	72 (943)	<.001
Women’s group or organization	38 (108)	62 (175)	12 (43)	88 (321)	<.001
Financial self-help group, or microfinance organization	40 (113)	60 (170)	11 (41)	89 (323)	<.001
Political, religious, caste, or ethnic	29 (81)	71 (202)	67 (244)	33 (120)	<.001
Group addressing slum rehabilitation, civic amenities, or entitlements	17 (47)	83 (236)	27 (98)	73 (266)	.002
Group addressing health or education	12 (35)	88 (248)	23 (84)	77 (280)	<.001
Any other type of community-based group or non-government organization	3 (9)	97 (274)	6 (21)	94 (343)	.120
Did any of the attended meetings discuss violence against women?	46 (131)	54 (152)	33 (120)	67 (244)	.001
Witnessed public gathering such as march, rally, or protest in past 12 months	46 (612)	54 (723)	62 (811)	38 (496)	<.001
Participated in march, rally, or protest in past 12 months	9 (124)	91 (1,211)	19 (242)	81 (1,065)	.510
*(If yes)* Issue raised by march, rally, or protest . . .^a^					
Women’s issues (e.g., anti-alcohol campaigns)	19 (23)	81 (101)	21 (52)	79 (190)	.402
Political, religious, or caste issues	74 (92)	26 (32)	78 (189)	22 (53)	.767
Slum rehabilitation, civic amenities, or entitlements	16 (20)	84 (104)	17 (42)	83 (200)	.275
Other issues	4 (5)	96 (119)	2 (5)	98 (237)	<.001
Did any of the attended public gatherings address violence against women?	39 (48)	61 (76)	36 (87)	64 (155)	.605

a Percentages add to more than 100% because respondents could have participated in more than one group or public gathering.

#### Perceived legitimacy of action against VAW

We found a range of opinions on the legitimacy of action against VAW (Table 2); 40–60% of women and men agreed that VAW was a common issue in their community and that it was serious. The majority disagreed that it was important for a husband to show his wife who was boss or that it was a wife’s obligation to have sex with her husband even if she did not feel like it. A majority disagreed that VAW was a private matter between those directly affected or that others outside the family should not intervene if a husband mistreated his wife. However, a majority also agreed that it was good for a wife to be afraid of her partner. Three quarters thought that men should take control in relationships and be head of the household, while 77–90% agreed that family problems should be kept within the family and domestic violence was a private matter. Women were slightly less likely than men to agree that wives were obliged to have sex with their husbands and that domestic violence was a private matter.

**Table 2.** Perceived Legitimacy of Action Against Violence Against Women.

	Women and trans people			Men			
	Generally agree	Generally disagree	Don’t know	Generally agree	Generally disagree	Don’t know	χ^2^-test of differences
Indicator	% (*n*)	% (*n*)	% (*n*)	% (*n*)	% (*n*)	% (*n*)	*p*
Concern about violence against women							
Violence against women is common in your community	40 (538)	58 (776)	2 (21)	40 (518)	59 (770)	1 (19)	.902
Violence against women is a serious issue for your community	60 (802)	40 (533)	0 (0)	60 (789)	40 (518)	0 (0)	.878
Women and girls are often harassed by men in your community^a^	55 (737)	45 (598)	0 (0)	57 (749)	43 (558)	0 (0)	.098
Acceptability of male power and control							
It is important for a man to show his wife who is the boss	37 (498)	63 (837)	0 (0)	35 (458)	65 (848)	0 (1)	.294
It is a wife’s obligation to have sex with her husband even if she doesn’t feel like it	40 (532)	59 (785)	1 (18)	41 (539)	56 (727)	3 (41)	.004
It is good for a woman to be a little afraid of her partner	61 (812)	39 (520)	0 (3)	58 (758)	42 (547)	0 (2)	.295
Men should take control in relationships and be the head of the household	71 (950)	29 (381)	0 (4)	75 (977)	25 (325)	0 (5)	.099
Acceptability of intervening in violence							
If a husband mistreats his wife, others outside the family should intervene	46 (616)	54 (719)	0 (0)	50 (657)	49 (646)	0 (4)	.012
Family problems should only be discussed with people in the family	88 (1,172)	12 (161)	0 (2)	90 (1,180)	10 (126)	0 (1)	.115
Domestic violence is a private matter to be handled in the family	77 (1,024)	23 (307)	0 (4)	84 (1,103)	15 (201)	0 (3)	<.001
Violence against women is a private matter between those directly affected	40 (539)	58 (780)	1 (16)	42 (543)	57 (743)	2 (21)	.524

a The original question was “How often are women and girls harassed by men in your community?” The answer options were “Every day,” “Sometimes,” “Rarely,” and “Never.” “Generally agree” indicates the respondent answered “Every day” or “Sometimes.” “Generally disagree” indicates the respondent answered “Rarely” or “Never.”

#### Perceived efficacy of action against VAW

Our data suggested an overall sense of efficacy (Table 3). Over 80% of respondents agreed their community could stop domestic violence, persuade the police to take action, or persuade families to support survivors of violence by working together. When asked about specific platforms for collective action, more than three quarters agreed that group discussion and peaceful demonstration would be effective, and a third of respondents even agreed that disruptive protest would be an effective means of stopping VAW. Even in terms of their own efficacy, over 80% felt they could make a personal difference to reducing VAW. However, on most indicators, men expressed greater confidence than women in the collective ability of their community to address VAW.

**Table 3.** Perceived Efficacy of Action Against Violence Against Women.

	Women and trans people			Men			
	Generally agree	Generally disagree	Don’t know	Generally agree	Generally disagree	Don’t know	χ^2^-test of differences
Indicator	% (*n*)	% (*n*)	% (*n*)	% (*n*)	% (*n*)	% (*n*)	*p*
Perceived efficacy to achieve specific outcomes							
In your neighborhood, you can stop domestic violence by working together	82 (1,096)	15 (205)	3 (34)	89 (1,161)	10 (125)	2 (21)	<.001
By working together, you can persuade the police to take action against domestic violence	85 (1,132)	13 (172)	2 (31)	88 (1,151)	10 (135)	2 (21)	.044
Together you can persuade families to support women facing domestic violence	95 (1,268)	4 (57)	1 (10)	97 (1,270)	3 (34)	0 (3)	.010
Perceived efficacy of specific interventions							
Do you think the following activities are effective in stopping violence against women . . .							
• Group meetings and discussions	87 (1,163)	10 (136)	3 (36)	88 (1,146)	10 (132)	2 (29)	.725
• Marches, rallies, or street theater	76 (1,020)	21 (275)	3 (40)	78 (1,021)	19 (251)	3 (35)	.568
• Sit-ins, blockages, or strikes	32 (426)	62 (829)	6 (80)	32 (420)	62 (816)	5 (71)	.825
Perceived personal efficacy							
A group of men would listen to you if you confronted them about their sexist behavior	32 (427)	63 (841)	5 (67)	44 (578)	51 (672)	4 (57)	<.001
You can help prevent violence against women in your community	91 (1,210)	9 (122)	0 (3)	94 (1,233)	5 (67)	1 (7)	<.001
You feel that your personal efforts can make a difference in reducing violence against women	83 (1,106)	14 (188)	3 (41)	90 (1,170)	8 (111)	2 (26)	<.001

#### Perceived norms for action against VAW

The majority of respondents indicated that family and community members would support them if they joined activities to stop VAW (Table 4). Over 70% of community members felt their families would approve of them joining such activities, would think such action prestigious, and would not find it opposed to their values or a waste of time. Seventy-three percent of women and 82% of men agreed that people in their neighborhood would approve of them joining activities to stop VAW. Only 6% felt it might be embarrassing to publicly state they were working toward preventing VAW. However, half of respondents—women, in particular—thought their neighbors might mock them for joining activities to stop VAW. Men were also more likely to expect family and community support in doing this than women.

**Table 4.** Perceived Norms for Action Against Violence Against Women.

	Women and trans people			Men			
	Generally agree	Generally disagree	Don’t know	Generally agree	Generally disagree	Don’t know	χ^2^-test of differences
Indicator	% (*n*)	% (*n*)	% (*n*)	% (*n*)	% (*n*)	% (*n*)	*p*
Perceived community support for individual action^a^							
People in your neighborhood approve of you joining activities to stop violence against women	73 (947)	21 (267)	6 (75)	82 (1,037)	13 (158)	5 (69)	<.001
People in your neighborhood would mock you for joining activities to stop violence against women	47 (601)	46 (592)	7 (96)	37 (462)	55 (699)	8 (103)	<.001
You would be embarrassed to say in public that you work to stop violence against women	6 (81)	93 (1,200)	1 (8)	6 (78)	93 (1,179)	1 (7)	.969
Perceived family support for individual action							
Your family members approve of you joining activities to stop violence against women	70 (929)	27 (365)	3 (41)	89 (1,168)	9 (118)	2 (21)	<.001
Your family members consider activities to stop violence against women opposed to their own values	32 (427)	65 (872)	3 (36)	19 (251)	79 (1,039)	1 (17)	<.001
Your family members consider spending 1 hr a week to stop violence against women a waste of your time	29 (393)	68 (903)	3 (39)	15 (198)	82 (1,078)	2 (31)	<.001
Your family members consider activities to stop violence against women prestigious work	68 (904)	27 (364)	5 (67)	85 (1,110)	11 (149)	4 (48)	<.001

a Sample sizes are smaller as questions on community support were introduced later into the survey.

### Qualitative Results

#### “Public” and “private” spaces

Qualitative data suggested explanations for variation in perceived legitimacy of action against VAW. Some community members saw domestic violence as a private matter to be borne by women with thoughtfulness and understanding (*sojh-samajh*). Speaking openly of such experiences was equivalent to speaking ill of one’s community and betraying the family’s honor (*izzat*). Instead, they reasoned, women should avoid letting things get worse and keep matters within four walls so that relations were not spoiled. Others had a more fluid conception of “public” and “private” space, and saw their neighborhood (*mohalla*) and alley (*galli*) as extensions of the domestic space, with the “real” outside being the wider settlement (*basti*); these made arguments to the effect that when everybody can hear the violence taking place next door, it is no longer a private matter for that family. Still others insisted that domestic violence was a social problem regardless of whether it was “public” or “private” and that prevention efforts benefited them collectively (*acche ke liye*). They pointed out that in serious or life-threatening cases of violence, one had to intervene. Younger women in particular questioned the silencing of domestic violence survivors due to shame (*sharm*) and fear (*dar*) and vocally supported intervention activities to address VAW.

#### Social and physical proximity

Qualitative data suggested explanations for perceived efficacy of collective action and expected social support for such action. Collective action and self-organizing were seen as vital to life in the neighborhoods and involved working with elected representatives to get services and infrastructure in place or resisting slum demolitions. Community members said that shared historical ties bound them together and created “unity” (*ekta*) while groups helped counter social inequality (*var-khali*) and care for the needy. They cited instances of standing up to local authorities, for example, when women demolished a wall which adversely affected the mobility of elderly and disabled community residents, after local police had been unresponsive to their complaints and even warned them “not to take law into their hands.” Residents specifically felt that their familiarity, close ties, and physical proximity to neighbors contributed to feelings of security. Some even articulated the view that perpetrators of public violence could be deterred by “beating them up and sending them packing.” Regarding domestic violence, residents felt they would notice immediately if there was noise among their neighbors or if someone’s door was closed during the day (given the overcrowded space in which residents live in slum communities, doors are only exceptionally closed; open doors allow for ventilation and socialization with neighbors).

#### Gendered social identities

Qualitative data suggested explanations for observed gender differences in participation in collective action. Some women worried that their group participation might invite negative judgments like “she is in a group!” or “she is being a leader!” alluding to an unseemly appetite for influence and power. Social identity considerations also led women to endorse control over married women by husbands and in-laws as a means of preserving the social status of themselves, their family, and community; as discussed above, making domestic violence public was seen by some as a betrayal of family and community honor. By contrast, male leadership was seen as largely “natural,” and many men already combined neighborhood activism with religious practice by participating in cleanliness drives, health camps, or festivals organized by local faith committees. However, men’s social identities rarely called for action to address VAW, and gender inequality was not a focus of these committees.

#### Reservations about taking action

Qualitative data also suggested reasons for why not all respondents felt confident about taking action. Community members feared retaliation from friends of perpetrators of violence, particularly in communities where substance abuse and crime were widespread and local criminal gangs used violence to silence opposition. Some worried that survivors of violence might themselves question their motives for trying to help in a “private matter.” Others thought community members might remain apathetic to the cause of preventing VAW due to domestic violence being normalized as “the story of every house” (*har ghar ki kahani*). A few men felt that managing one household was already complex enough without having to address neighbors’ domestic disputes. Such protracted entanglements might endanger mutually beneficial relations with neighbors, take time, distract them from the need to pursue daily wage labor, and complicate their own domestic life if their family members disagreed with them. Attending to others’ domestic disputes to the neglect of own affairs might thus result in one’s “daily bread (*roti-sabzi*) burning to ashes!”

## Discussion

We present, to our knowledge, the first study to systematically map social and psychological readiness for collective action against VAW in a low- and middle-income context. Such a mapping is important as attitudes to VAW vary enormously with context. For example, the proportion of respondents who agreed that wife-beating was justified if the wife had burned the food ranged from 0.2% in Cyprus to 60% in Jordan (Waltermaurer, 2012). Our findings suggested that urban informal settlements are heterogeneous communities in which attitudes endorsing gender inequality and violence can coexist with significant enthusiasm and support for collective action against VAW. Overall, communities might be well prepared to engage with a community mobilization intervention to address VAW in our context.

Our findings add nuance to portrayals of urban informal settlements as sites of anomie, hopelessness, and crime, in which ideas of self-help and self-organization are but an “illusion” (Campbell & Mzaidume, 2001; Davis, 2006; Kongelf et al., 2015). An evaluation of a gender-transformative program to reduce domestic violence in South African urban informal settlements concluded that low social cohesion had impeded efforts at community mobilization, as most residents had neither feelings of belonging, nor investment in their community, nor plans to stay long term (Hatcher et al., 2020). An ethnography in Mumbai, India, found that population density and proximity of neighbors only imbued an element of spectacle to incidents of domestic violence, leading neighbors to watch, even enjoy, but not intervene in incidents (Ghose et al., 2008). A study in Delhi found that women were hesitant to call on neighbors for help, fearing backlash from their family members and judgment from their neighbors, whom they distrusted (Snell-Rood, 2015).

By comparison, rural environments are sometimes depicted as places of extensive social networks, close-knit neighbor relations, and abiding economic cooperation (De Silva et al., 2006; Gregson et al., 2011). Our findings showed that residents in informal settlements in Mumbai equally saw themselves as socially cohesive units with a history of cooperation over such issues as access to services and infrastructure and resistance to slum demolitions, in which participation in community groups, marches, or mass gatherings was relatively common. While our study confirmed the presence of patriarchal attitudes—such as the idea that women should endure domestic violence as part of their duty of care for the family—large proportions of women and men did support collective action to address VAW.

Some of the differences in findings between ours and previous studies might be due to methodology, as previous studies of residents’ willingness to take action against VAW have been based on a small sample of respondents which may not have reflected the diversity of the wider settlement. Surveys of attitudes to VAW in India have found that less than half of men and women at national and subnational levels endorse justifications for wife-beating (International Institute for Population Sciences [IIPS] & ICF, 2017). Some of the difference may also be due to secular change. A study in rural Maharashtra ascribed a sustained 40% drop in acceptance of wife-beating between 2012 and 2013 after widespread media coverage of a gang rape in Delhi (Nirbhaya) that mobilized nationwide protests against VAW (Shakya et al., 2017). Nonetheless, the relative agreement between qualitative and quantitative results adds plausibility to our findings.

The coexistence of support for action to address VAW with patriarchal attitudes presents an apparent paradox. We propose three hypotheses to resolve this. First, individual differences between community members might have resulted in some community members endorsing action to address VAW, while others were opposed to it. For example, our qualitative data indicated a generational divide with younger women being more willing to challenge gender inequality than older women. Second, subtle differences in the acceptability of different forms of violent behavior might have led some respondents to hold both views simultaneously (Schuler et al., 2011). Some might have thought controlling behavior acceptable, but not physical violence. Others might have felt “light” physical violence—such as a slap—was acceptable, but not “severe” violence. Third, some respondents might have seen violence as a necessary, but unfortunate tool to control the behavior of women and thus endorsed efforts to keep such “necessary evils” to a minimum (Go et al., 2003). Our data did not allow us to establish a dominant motive, but future researchers should explore the extent to which the above explanations account for variation in support for action to address VAW.

Our findings have limitations. Our need to map an unobservable such as readiness to take collective action made it necessary to rely on self-report. As respondents knew our organization was committed to ending VAW, they may have been motivated to position their communities favorably so as to receive services and support (Skovdal et al., 2017). Randomized comparison of survey responses for men and women who were given either a self-administered or face-to-face interview showed little overall evidence of social desirability bias (Gram et al., 2020). Baseline readiness to take collective action to address VAW may also not necessarily be predictive of long-term engagement with public health interventions. Community mobilization can create complex, dynamic trajectories of behavior, in which agents continually adapt to their environment and history of events. For example, high levels of perceived efficacy of action against VAW may reflect overconfidence due to a lack of experience with such efforts (Dunning, 2011). Finally, the majority of attendants at micro-planning meetings were women, which prevented us from fully saturating themes concerning men’s attitudes to action to prevent VAW.

Nevertheless, we believe our findings contain useful lessons for future research and policy. Community mobilization interventions to address VAW through collective action are gaining traction among practitioners and policymakers, but their implementation has not always succeeded (Mannell et al., 2019). Intervention researchers have ascribed lack of impact to contextual challenges (Chatterji et al., 2020; Hatcher et al., 2020), but have hitherto not mapped out readiness to tackle VAW at baseline. We show that such a mapping does not uniformly paint informal settlements as places where capacity for collective action needs to be built from scratch. Future researchers might similarly map their own context at baseline, enabling us to compare systematically the impact of readiness for action on intervention impact.

In our context, a strictly phased approach (Michau, 2007) may not be optimal, as some individuals in the community may already be aware of the problem and ready to take action at baseline. Implementers could seek out such actors during community entry and engage them in early tasks: persuading coresidents to join the cause, finding other sympathetic community members, and raising awareness about the need to respect women’s rights. In time, early volunteers could evolve into community organizers with responsibilities of their own for mobilizing collective action. While this is to some extent the modus operandi of existing interventions (Daruwalla, Jaswal, et al., 2019), future researchers could explore introducing such elements earlier to build rapid capacity for collective action and galvanize communities around early success. This opens up avenues for policymakers and practitioners to treat communities as less vulnerable and more capable of changing situations and problems that affect them.
